# Short-Term Probiotic Administration Increases Fecal-Anti *Candida* Activity in Healthy Subjects

**DOI:** 10.3390/microorganisms7060162

**Published:** 2019-06-03

**Authors:** Massimiliano De Angelis, Carolina Scagnolari, Alessandra Oliva, Eugenio Nelson Cavallari, Luigi Celani, Letizia Santinelli, Giuseppe Pietro Innocenti, Cristian Borrazzo, Giancarlo Ceccarelli, Vincenzo Vullo, Gabriella d’Ettorre

**Affiliations:** 1 Department of Public Health and Infectious Diseases, University of Rome-Sapienza, Rome 00185, Italy; massimiliano.deangelis@yahoo.com (M.D.A.); alessandra.oliva@uniroma1.it (A.O.); eugenionelson.cavallari@uniroma1.it (E.N.C.); luigi.celani@uniroma1.it (L.C.); letizia.santinelli@uniroma1.it (L.S.); giuseppepietro.innocenti@uniroma1.it (G.P.I.); cristian.borrazzo@uniroma1.it (C.B.); vincenzo.vullo@uniroma1.it (V.V.); gabriella.dettorre@uniroma1.it (G.d.E.); 2 Department of Experimental Medicine- Virology section, University of Rome-Sapienza, Rome 00185, Italy; carolina.scagnolari@uniroma1.it

**Keywords:** *Candida*, fecal water, probiotics, Vivomixx^®^, *VSL#3^®^*

## Abstract

Background: *Candida albicans*’ ability to evade host immune responses represents a serious threat for vulnerable patients. Objectives: To investigate if (1) feces from healthy subjects exert anti-*Candida* activity; (2) fecal anti-*Candida* activity is modified by probiotic administration and (3) different probiotic differently modulate anti-*Candida* activity. Patients and methods: Feces from healthy donors were analyzed before and after seven days of dietary supplementation with two different probiotic formulations (VSL#3^®^; Vivomixx^®^). *Candida albicans* was cultured with decreasing concentrations of diluted feces, obtained before and after the treatment period. The relationship between anti-*Candida* activity of feces, interferon-α, anti-interferon-α antibodies and the expression of MxA, ISG15 and IFNAR1 was also evaluated. Results: Feces obtained prior to probiotic intake and feces collected after supplementation with VSL#3^®^ did not affect *Candida albicans* growth. On the contrary, a 3log_10_ inhibition of *Candida* development was observed after Vivomixx^®^ intake. Interferon-α played a role in the inhibition of *Candida* growth. Conclusion: Fecal anti-*Candida* activity was not observed prior to probiotic supplementation. Seven days of administration of Vivomixx^®^ increased fecal anti-*Candida* activity, the same effect was not observed after intake of VSL#3^®^. The probiotic-induced anti-*Candida* activity seems to be related to an increased local production and release of interferon-α. Clinical trials are needed to determine if a short pretreatment with specific probiotic formulations may increase anti-*Candida* defenses in patients at risk.

## 1. Introduction

*Candida albicans* (*C. albicans*) is a natural inhabitant of the gut and at the same time may behave as an aggressive pathogen causing life-threatening disease [1]. The human immune system has developed multiple strategies to fight fungal infections, but at the same time *Candida* spp. has developed various survival mechanisms, such as the ability to evade recognition by host immune cells [2]. The host anti-*Candida* defense system is also reinforced by some gut-resident bacteria, which are capable of restraining the growth of the fungus [3]. Nevertheless, under certain circumstances the equilibrium between host activities, bacterial activities, and fungal activities may be disrupted and turn into a pathological state [4,5]. For example, studies conducted on patients with chronic intestinal inflammatory bowel diseases (IBD) have shown a link between inflammation and overgrowth of *C*. *albicans* [5]. In addition, patients with cirrhosis may develop spontaneous *C. albicans* peritonitis, and in subjects with irritable bowel syndrome (IBS) associated with visceral hypersensitivity an intestinal fungal dysbiosis has been reported [6].

It is a common practice to use lactic acid bacteria (principally *Lactobacillus* and *Bifidobacterium* genera) with antifungal properties for biopreservation of food [7] and it is well known that a healthy vaginal microbiota is generally dominated by *Lactobacillus* spp. The protection provided by the lactobacilli has been generally attributed to pH control, the release of metabolites with antifungal activity (particularly fermentative metabolites such as lactic acid, acetic acid and succinic acid) [7,8], or even to an interference with the fungus cell cycle [9]. Despite that those protective mechanisms remain poorly understood, recent evidences showed that the type I IFN pathway plays a central role in the immune responses to *C. albicans* in humans and that components of the gut microbiota can have distal effects on responses to a number of infections through the modulation of type I IFN [10,11].

Accordingly, the primary scope of our research was to test the hypothesis that upon ingestion, probiotic formulations without any demonstrated anti-*Candida* activity in vitro may nevertheless increase the anti-*Candida* activity of the feces by changing the equilibrium among the species present in the gut and/or induce the host to produce and release molecules with anti-*Candida* activity. The secondary endpoint were (I) to assess whether this effect was influenced by differences in the probiotics used and (II) to evaluate if anti-*Candida* activity was linked with the modulation of type I IFN.

## 2. Materials and Methods

### 2.1. Demographic Characteristics of the Participants.

**Figure 1:**
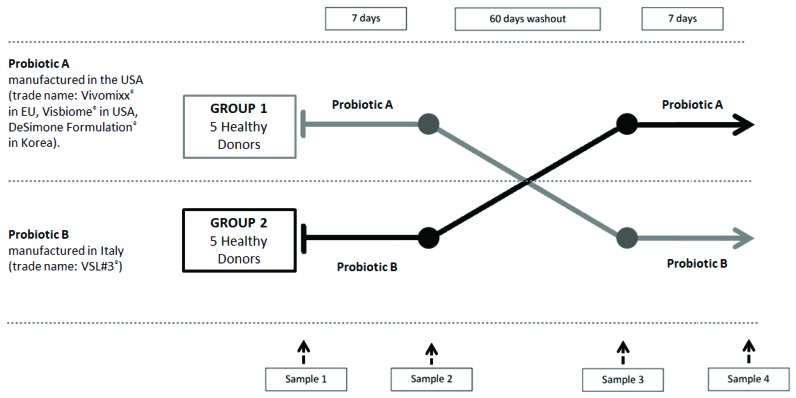
Protocol flow-chat.

The present study was a cross-over, pilot study and included 10 healthy individuals divided in 2 groups (group 1 and 2). All subjects were enrolled at the Clinic of the Department of Public Health and Infectious Diseases of University of Rome “Sapienza”. The study protocol was approved by the internal committee of the Department of Public Health and Infectious Diseases of “Sapienza” University of Rome and by the Ethics Committee of Policlinico Umberto I Hospital, Rome (Rif. C.E. 2970). All participants agreed by signing a written informed consent form to be treated for a week with a probiotic, then to observe a period of eight weeks of wash out, and then to be treated again with a formulation different from the one they were treated previously. We collected 40 samples of feces and for each subject four samples, two before and two after the treatments (Figure 1).

All study participants were healthy Caucasian men (health donors—HD) with a median age of 35 years [Interquartile Range (IQR) 22–53 years]. Exclusion criteria were patients with (1) a known allergy or intolerance to the product, (2) diarrhea, (3) a history of or current inflammatory diseases of the small or large intestine, (4) any past or current systemic malignancy, (5) previous or a current drug addiction and (6) past *Candida spp*. infections. All subjects enrolled in the study showed a negative microscopic examination of the stool, a negative bacterial stool culture and a negative result for the research of pathogenic viruses in the stool.

Group 1 started with a 7 days course of the probiotic A manufactured in the USA (trade name: Vivomixx^®^ in EU, Visbiome^®^ in USA, DeSimone Formulation^®^ in Korea) and containing *Lactobacillus plantarum* DSM24730, *Streptococcus thermophilus* DSM24731, *Bifidobacterium breve* DSM24732, *Lactobacillus paracasei* DSM24733, *Lactobacillus delbrueckii subsp. bulgaricus*^±^ DSM24734, *Lactobacillus acidophilus* DSM 24735, Bifidobacterium longum* DSM24736 and Bifidobacterium infantis* DSM24737 (^±^: recently reclassified as *Bifidobacterium lactis*; *: recently reclassified as *Lactobacillus helveticus*). After the washout period (60 days) start a second 7 days course of the probiotic B, manufactured in Italy (trade name: VSL#3^®^), containing *Streptococcus thermophilus* BT01, *Bifidobacterium breve* BB02, Bifidobacterium longum* BL03, Bifidobacterium infantis* BI04, *Lactobacillus acidophilus* BA05, *Lactobacillus plantarum* BP06, *Lactobacillus paracasei* BP07 and *Lactobacillus delbrueckii subsp. bulgaricus*^±^ BD08 (^±^: recently reclassified as *Bifidobacterium lactis*; *: recently reclassified as *Lactobacillus helveticus*). Group B started with a 7 days course of the probiotic B and after the washout period (60 days) start a second 7 days course of the probiotic A.

For all donors the supplementation dose was three sachets/day for a total of 1350 billion bacteria day for seven days. This dosage was in the range of the daily amount recommended by the marketers and previously reported in the literature.

Enrollment, fecal sample collection and additional tests were all conducted during the same time period for both HD receiving Vivomixx^®^ and HD receiving VSL#3^®^.

### 2.2. Microbiological Analyses

For the microbiological analyses, the *C. albicans* reference strain (ATCC 14053) was used [12]. The strain was stored on a cryovial bead preservation system (Microbank; Pro-Lab Diagnostics, Richmond Hill, Ontario, Canada) at −80 °C. A *C. albicans* inoculum was prepared by spreading one cryovial bead on a blood agar plate and Sabouraud dextrose agar (Liofilchem S.r.l. Via Scozia, Zona Industriale 64026, Roseto degli Abruzzi (TE)) and incubated overnight at 37 °C. One colony was re-suspended in a 5 mL Sabouraud dextrose broth and incubated at 37 °C without shaking. Overnight cultures were then adjusted to a turbidity of 2 McFarland, corresponding to ≈1 × 10^8^ CFU/mL. The anti-*Candida* activity was evaluated by using the macrobroth dilution in a final volume of 1 mL.

### 2.3. Anti-Candida Activity of Probiotic Formulations

Probiotic formulations were dissolved in 20 mL of NaCl 0.9% solution. After 30 min of incubation at room temperature, the solution was centrifuged at 4500 rpm for 15 min and 450 µL of the collected surnatant were added to 450 µL of Mueller-Hinton broth (MHB). A final *C. albicans* inoculum of 5 × 10^5^ CFU/mL was added to tubes containing two-fold serial dilutions of MHB plus probiotic surnatant and incubated at 37 °C for 24 h. Minimal inhibitory dilution (MID) and minimal bactericidal dilution (MBD) were defined as the lowest dilution that completely inhibited visible growth and ≥99.9% (i.e., ≥3log_10_ CFU/mL) reduction of the initial *C. albicans* count after 24 h of incubation, respectively [13].

### 2.4. Anti-Candida Activity of Fecal Water

Stools (200 mg) collected at time 0 and time 1 (before and at the end of 7-day probiotic supplementation, respectively) were dissolved in 2 mL of phosphate buffered saline (PBS), with a final concentration of 100 mg/mL, vortexed and then assayed for anti-*Candida* activity (fecal water). A final *C. albicans* inoculum of 5 × 10^5^ CFU/mL was added to tubes containing two-fold serial dilutions of the Mueller Hinton broth (MHB) plus fecal water at the final concentration of 50 mg/mL and incubated at 37 °C for 24 h. A total of 20 μL was then plated on the Sabouraud agar and colonies were counted after an additional 24 h of incubation. Minimal fungicidal dilution (MFD), together with the correspondent concentration of fecal water (mg/mL), was defined as the fecal water dilution obtaining ≥99.9% (i.e., ≥3log_10_ CFU/mL) reduction of the initial fungal count after 24 h of incubation. The limit of detection was 50 CFU/mL. Each experiment was run in duplicate.

### 2.5. Anti-Candida Activity of IFN-a

To assess the potential role of *IFN-a* against *C. albicans*, two-fold serial dilutions of *IFN-a* (starting from 1000 IU/mL) plus MHB containing a final 5 × 10^5^ CFU/mL of fungal inoculum were tested, as described above. In a subsequent experiment, antibodies against *IFN-a* (50 μL) were added to fecal water (50 μL, with a final concentration of 25 mg/mL) collected at T1 from HD receiving Vivomixx^®^ and MHB (100 μL). After 24 h incubation with 5 × 10^5^ CFU/mL of *C. albicans*, MFD was calculated as previously described.

Quantitative real-time PCR for MxA, ISG15 and IFNAR1 was carried out with the LightCycler 480 instrument (Roche, Basel, Switzerland). Briefly, the total RNA was extracted from A549 cells using the RNeasy Plus Universal Tissue Mini Kit (Invitrogen, Carlsbad, CA, USA) and reverse transcribed using the High Capacity cDNA Reverse Transcription Kit (Applied Biosystems, Foster City, CA, USA), according to the manufacturer’s protocol. Primers and probes for each gene were added to the Probes Master Mix (Roche, Basel, Switzerland) at 500 nM and 250 nM, respectively, in a final volume of 20 μL. The housekeeping gene β-glucuronidase was used as an internal control. Gene expression values were calculated by the comparative Ct method using the equation 2-ΔΔCt (ΔΔCt method) [14]. The list of primers and probes is as follows: ISG15 (Forward: 5′-TGGCGGGCAACGAATT-3′, Reverse: 5′-GGGTGATCTGCGCCTTCA-3′, Probe: 5′-[6 carboxyfluorescein(6FAM)]TGAGCAGCTCCATGTC [tetramethylrhodamine(TAM)]-3′); MxA: (Forward: 5′-CTGCCTGGC AGAAAACTTACC-3′; Reverse: 5′-CTCTGTTATTCT CTGGTGAGTCTCCTT-3′; Probe: 5′-[6FAM]CATCAC ACATATCTGTAAATCTCTGCCCCTGTTAGA[TAM]-3′) and β-glucuronidase: (Forward: 5′-TCTGTCAAGGGCAGTAACCTG-3′, Reverse: 5′-GCCCACGACTTTGTTTTCTG-3′, Probe: 5′-(6FAM)TATGTCTTTCGATATGCAGCCAAGTTTTACCG3′(TAM)-3′). Primers and probe sequences used for IFNR1 (Hs. PT.58.25402720.g) were purchased from Integrated DNA Technologies (IDT), Iowa, USA. Each experiment was run in triplicate.

### 2.6. Statistical Analysis

Results were expressed as mean ± standard deviation (SD) or median (min–max), as appropriate. Continuous data (i.e., log_10_ CFU/mL) were analyzed with the non-parametric Mann–Whitney test. ANOVA test was used to compare the means of more than two groups. Statistical analyses were performed using STATA 9 software (STATA corp. LP, College Station, TX, USA) and GraphPad Prism version 7 for Windows (GraphPad Software San Diego, CA 92108, USA), as appropriate. All statistic tests were two-tailed and a *p*-value <0.05 was considered statistically significant.

## 3. Results

All the subjects tolerated the probiotic treatment without any adverse effects, with the exception of one patient who exhibited labialis *H. Simpl*ex on the third day of treatment with VSL#3^®^. The fecal samples of this individual were not evaluated.

After incubation at 37 °C for 24 h, the morphological state of *C. albicans* culture was checked: In the cultures performed, *C. albicans* did not undergo yeast-to-hyphae transition, as in previous studies [15]. As for the antimicrobial activity of probiotic formulation (surnatant), no inhibition of *C. albicans* was observed, with fungal growth (>10^8^ CFU/mL) observed at the lowest dilution (1:2).

**Figure 2:**
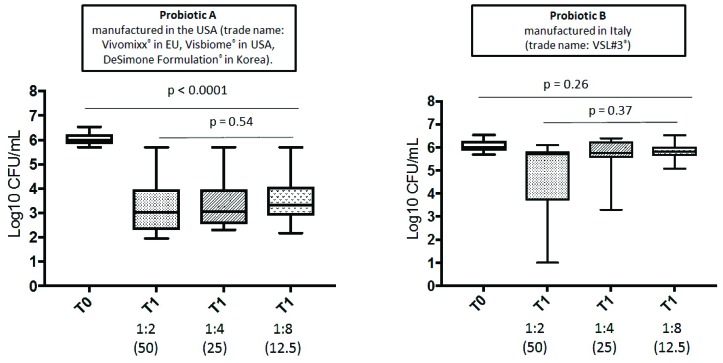
Candia albicans growth inhibition effect observed at different dilutions of fecal water in Vivomixx and VSL#3 supplemented participants.

At time 0, the fecal water samples (total number 20) of all volunteers did not inhibit *Candida* growth in Petri plates (6.070 ± 0.267 and 6.046 ± 0.263 log_10_ CFU/mL for subjects receiving VSL#3^®^ and, Vivomixx^®^, respectively). Conversely, at time 1 (total number 19 samples), the fecal water samples (*n* = 10) from HDs receiving Vivomixx^®^ showed markedly reduced *Candida* growth compared to the starting inoculum (log_10_ CFU/mL 5.732; log_10_ CFU/mL 3.312 ± 1.345, 3.439 ± 1.253, 3.589 ± 1.183, at 1:2 (50 mg/mL), 1:4 (25 mg/mL), and 1:8 (12.5mg/mL) dilutions, respectively) while the anti-*Candida* activity of fecal water samples (*n* = 9) from HDs receiving VSL#3^®^ was barely detectable (log_10_ CFU/mL 4.687 ± 1.99, 5.621 ± 0.929, 5.822 ± 0.390 at 1:2 (50 mg/mL), 1:4 (25 mg/mL), and 1:8 (12.5 mg/mL) dilutions, respectively; Figure 2).

When *IFN-a* was added to the fecal water samples previously devoid of anti-*Candida* activity (HDs receiving VSL#3^®^), the growth of *Candida spp.* was inhibited up to a 1:32 dilution. The addition of *IFN-a* to the fecal water of HDs receiving Vivomixx^®^ did not further increase anti-*Candida* inhibitory potential while the addition of *IFN-a* antibodies to these samples resulted in a high fungal growth at all tested dilutions, confirming a relationship between fecal anti-*Candida* activity and the presence of *IFN-a*. No modification was observed when *IFN-a* antibodies were added to HDs receiving VSL#3^®^.

**Figure 3:**
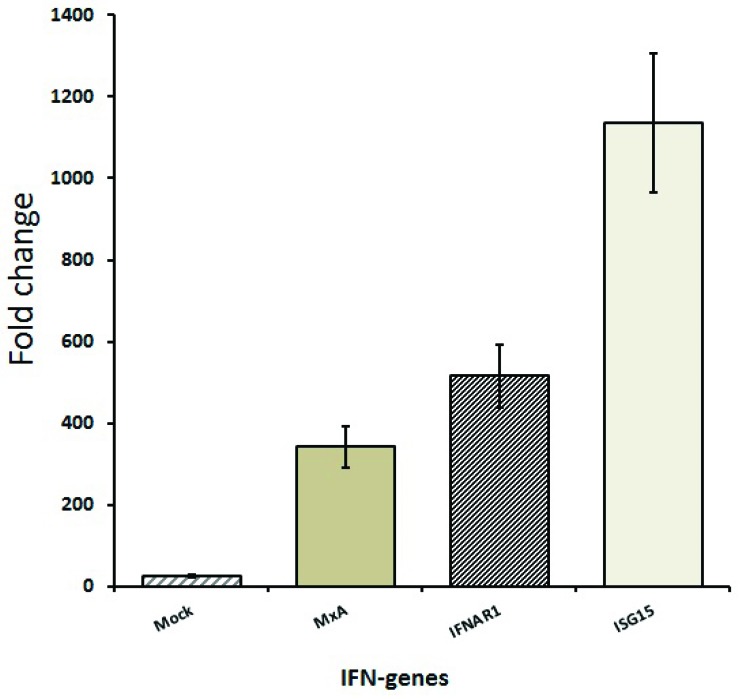
Human mycovirus resistance protein 1 (MxA), Interferon-α/β receptor α chain gene (IFNAE) 1 and interferon stimulated gene 1 (ISG15) production from A549 cells after incubation with HD1 fecal water.

Moreover, a strong induction of human MxA protein, interferon-stimulated gene 15 (ISG15), and interferon-α/β receptor α chain gene (IFNAR1) was detected when the A549 cells, usually employed in viral research and associated protein expression changes, were incubated with fecal water from HD1 (Figure 3).

As a whole, these results suggest that the presence of *IFN-a* in fecal water is a determinant for *C. albicans* inhibition and that one formulation, i.e., Vivomixx^®^ is much more effective in inducing a protective *IFN-a*-mediated local immune response. The complete characterization of the fecal samples, including measurement of *IFN-a* as well as the levels of the fecal volatile fatty acids and the metabolomics is in progress.

## 4. Discussion

Our approach obviously did not reflect how effective the anti-*Candida* defense system is as a whole, but only that portion related to the excreted fecal mass. The fact that the subjects enrolled in our study were all healthy and did not develop any *Candida* infection during the following four months proves that their anti-*Candida* defenses were efficient at the moment of enrollment. However, we have shown that the administration for just one week of a specific probiotic product that is per se unable to inhibit in vitro *Candida* growth was able to modify the capability of the gut flora to inhibit the growth of *Candida*. This increased anti-*Candida* activity can be considered irrelevant in healthy subjects where the immune system and other components function efficiently, but may be relevant in other clinical situations. For example, if we accept the hypothesis that recurrences of Crohn’s disease are somehow related to an overgrowth of *Candida*, then a proper probiotic treatment could be a contributing factor to the maintenance of clinical remission [16]. Another situation, still in the research phase, is schizophrenia, where a relationship between the severity of the disease and antibody response to *Candida* has been observed.

Since *Candida* is a frequent colonizer our data may have the potential to impact many people. Chronic administration of anti-fungals is not a feasible option and therefore any “safe” alternative to prevent *Candida* spp. infections, such as probiotics, should be taken in due consideration. It is possible to hypothesize a short pre-treatment with probiotics to increase the anti-*Candida* defenses in patients who will undergo immunodepression due to the evolution of their disease, such as cirrhosis, or induced by drugs or irradiation, such as leukemia [17,18,19].

At the same time, we also demonstrated that not all probiotic preparations are suitable in inducing an anti-*Candida* activity in the feces. The subjects who ingested VSL#3^®^ did not show any significant variation in anti-*Candida* fecal activity, contrary to subjects who were treated with Vivomixx^®^. Notably, this finding was reconfirmed when subjects were shifted to the other product, since our study was a cross-over study. We used two products that are apparently similar in composition but different in manufacturing process [20]. Moreover, we do not know if the two products tested by us have the same bacterial composition. The product labels show a similarity in terms of bacterial species, but according to supplementary Table 3 of a recent publication of Douillard et al. [21], VSL#3^®^ seems to contain seven strains. Vivomixx^®^ contains eight strains and it is possible that its capability to induce anti-*Candida* activity depends on the presence of a different number of strains in different proportions [22].

To summarize, three observations are relevant. The first one is the fact that the induction of anti-*Candida* activity is rather quick, after just one week or even less of probiotic supplementation. This suggests that the observed anti-*Candida* activity in fecal water is a consequence of a probiotic-induced shift of the equilibrium among the different bacterial populations present in the gut in favor of the species with anti-*Candida* activity. This short timeframe for induction also confirms the feasibility of our approach in many clinical situations in order to prevent *Candida* spp. infection in patients at risk. Previously, other works have already shown that short periods of high dose probiotic administration are able to induce adequate intestinal colonization and clinical effects [23,24].

The second relevant observation is that VSL#3^®^ was ineffective compared to Vivomixx^®^, which reconfirms that if the probiotic formulations are produced by different producers that use different strains, methods and media to ferment the bacteria, they will have a different clinical efficacy, as recently suggested [25]. Unfortunately, very few people pay due attention to what is reported on the product label since they are not aware that the probiotic market is still poorly regulated and what may look similar is quite different in reality.

The third observation is related to a different induction of the host cytokines by the two formulations. Our preliminary results showed that the reduction of almost 3log_10_ CFU/mL in the growth of *Candida* in the fecal water of subjects who took Vivomixx^®^ was associated with an increased release of *IFN-a* at T1, and that this inhibitory action was lost after the addition of anti-*IFN-a* antibodies. On the basis of these data, we hypothesized that specific components and/or metabolites of the gut microbiota of subjects treated Vivomixx^®^ contribute to the increase the anti-*Candida* activity of the feces by enhancing IFN signaling.

Previously, it has been shown that *Lactobacillus reuterii* changes its metabolism in response to *Candida* spp. overgrowth in the gut, ceasing to use sugar and starting to consume amino acids to produce an inducer of Interleukin 22, a substance, which controls the proliferation of *Candida* spp. and protects the gut wall from inflammation [26,27,28]. Since a modulation of the tryptophan/serotonin pathway associated with a modification of the immune response has already been shown in human immunodeficiency virus-positive patients treated with Vivomixx^®^, we cannot exclude the involvement of this pathway in the healthy volunteers treated with Vivomixx^®^ [29].

The major limitation of this study was the small sample size and larger studies should be performed to confirm and extend these findings. Nevertheless, it is the first time that a cross-over study in healthy subjects evidenced that administering a short course of a high concentration multistrain probiotic formulation had the potential to modify the resident gut flora to increase anti-*Candida* activity.

In conclusion a number of preliminary clinical studies suggested that probiotics might play a role in the reduction of *Candida* colonization on human mucosal surfaces, potentially reducing the risk of not only oral, vaginal and intestinal colonization, but also the incidence of invasive fungal infections [30]. Our data not only confirmed this suggestion providing a basis for a larger study, but also clarified that not all probiotic formulations are suitable in situations where *Candida* spp. may play a pathogenic role.
